# Lifestyle-based community exposure and self-rated health: a multilevel analysis of neighborhood disadvantage in Urban China

**DOI:** 10.3389/fpubh.2026.1785410

**Published:** 2026-04-10

**Authors:** Jinfu Xu, Yanjun Wu, Xiaochang Lv, Liyuan Yao, Yee Cheng Kueh, Garry Kuan, Liying Yao

**Affiliations:** 1 Journal Center, Guangzhou Sport University, Guangzhou, China; 2 School of Physical Education, Guangzhou University, Guangzhou, China; 3 Faculty of Arts, Design & Architecture, University of New South Wales, Paddington, NSW, Australia; 4 Biostatistics and Research Methodology Unit, School of Medical Sciences, Universiti Sains Malaysia, Kubang Kerian, Kelantan, Malaysia; 5 Exercise and Sports Science Programme, School of Health Sciences, Universiti Sains Malaysia, Kubang Kerian, Kelantan, Malaysia

**Keywords:** latent class analysis, lifestyle exposure, neighborhood disadvantage, public health, self-rated health

## Abstract

**Background:**

Neighborhood socioeconomic disadvantage is a well-established determinant of population health, yet residents living in the same communities often experience unequal health outcomes. Such heterogeneity may reflect differences in lifestyle-based exposure to local environments. However, evidence on whether and how this exposure patterns modify neighborhood health effects remains limited, particularly in rapidly urbanizing contexts such as China. This study examined whether lifestyle-based community exposure moderates the association between neighborhood concentrated disadvantage and self-rated health among urban residents.

**Methods:**

Data were obtained from the 2018 China Labor-force Dynamics Survey, including 2105 urban residents nested within 133 communities. Neighborhood concentrated disadvantage was constructed using principal component analysis of community-level socioeconomic indicators. Community exposure patterns were identified via latent class analysis based on five lifestyle-related behaviors: smoking, alcohol consumption, regular physical exercise, neighborhood reciprocity, and community participation. Multilevel logistic regression models were used to estimate the association between neighborhood disadvantage and self-rated health and to test cross-level interaction effects, adjusting for individual sociodemographic and socioeconomic characteristics.

**Results:**

Three distinct lifestyle-based community exposure patterns were identified: health-oriented (13.37%), mixed (21.11%), and high-risk isolated (65.53%). Higher neighborhood concentrated disadvantage was significantly associated with lower odds of reporting good self-rated health. Compared with the health-oriented reference group, residents in the mixed (risk/part) class reported significantly poorer self-rated health (*p* < 0.01). Critically, cross-level interaction terms between neighborhood disadvantage and lifestyle typologies were non-significant, indicating that structural and behavioral factors operate as independent, additive determinants of health rather than as a buffering system.

**Conclusions:**

In contemporary urban China, neighborhood socioeconomic disadvantage and individual lifestyle patterns function as two parallel and independent risk pathways shaping health outcomes. Reducing urban health disparities therefore requires both structural neighborhood interventions and targeted behavioral strategies, rather than assuming that healthy lifestyles can offset the effects of severe spatial deprivation.

## 1 Introduction

Neighborhood context is increasingly recognized as a key socioecological factor influencing health (1). Although the “social causation” of individual socioeconomic status is well established (2), the structural influences of residential settings, commonly referred to as “neighborhood effects,” remain central to epidemiological and sociological research. A substantial body of research in Western countries has demonstrated that community-level socioeconomic disadvantage significantly impairs adult health outcomes and contributes to health inequalities (3–5).

China's rapid urbanization offers a unique lens for re-examining these dynamics. The commodification of housing has intensified social-spatial differentiation, creating distinct residential stratification (6). Unlike in Western contexts, Chinese urban communities serve dual functions: they are both living spaces and key grassroots units within the state governance system, acting as the interface between the state and society (7, 8). Despite this crucial structural significance, existing Chinese research has primarily focused on whether neighborhood effects exist (the average impact), often treating communities as static “containers” that uniformly affect all residents.

This “uniformity assumption” overlooks the complexity of interpersonal environmental interactions (9). Even in identical community settings—sharing the same infrastructure, safety levels, and demographic profiles—residents' participation patterns vary widely (10). Sampson (2019) note that neighborhood research stands at a crossroads: to understand how environments function, we must move beyond average effects and examine heterogeneity (11). This heterogeneity is primarily driven by “exposure”—the specific “dose” of community characteristics an individual actually encounters, shaped by their lifestyle and activity spaces. The prevailing “buffering hypothesis” posits that adopting a healthy lifestyle can effectively offset the structural harm inflicted by disadvantaged neighborhoods. However, against the backdrop of China's rapid urbanization and profound lifestyle transitions, we argue that these structural and behavioral forces likely operate on parallel tracks.

From a social-ecological perspective, the impact of the macro-environment on individual health is conditional on the degree of engagement between the resident and their community (12, 13). Grounded in this framework, the present study pursues three analytical objectives. First, we operationalize community lifestyle as a multidimensional exposure indicator using latent class analysis, identifying distinct patterns of resident–community interaction. Second, we examine whether neighborhood disadvantage and these lifestyle classes exert independent effects on self-rated health. Third, we formally adjudicate between two competing theoretical paradigms—the “buffering hypothesis,” which posits that healthy lifestyles can mitigate structural disadvantage, and the “parallel tracks hypothesis,” which holds that these forces operate independently and additively. Data from the 2018 China Labor-Force Dynamics Survey (CLDS) are used for all analyses.

## 2 Theoretical background and hypotheses

### 2.1 The structural constraint: neighborhood disadvantage and health

The social-ecological model holds that health is shaped by nested layers of influencing factors, with the community serving as a critical meso-level (14). Existing literature indicates that community disadvantage—typically manifested as concentrated poverty, high unemployment, and household instability—consistently correlates with poor self-rated health, chronic diseases, and mortality (15–17). Its mechanisms of action primarily manifest in two dimensions: material deprivation (service scarcity, and weak infrastructure) and psychosocial stress (crime, disorder, and lack of social control) (18, 19).

In the Chinese context, these mechanisms manifest in unique ways. While wealthy communities typically offer better access to healthcare resources and promote physical well-being, social dynamics may differ. Some studies reveal the “Shanghai Paradox”: older adults in low socioeconomic status communities report higher social cohesion, which mitigates depressive symptoms (10). Structurally, however, the disadvantages of concentrated communities in China are often linked to outdated infrastructure and limited public services. Despite individuals' potential to choose their residential communities autonomously, Levy argues that community structural mechanisms operate independently of personal choice, thereby creating persistent “spatial inequality” (20). We therefore propose: Hypothesis 1 (H1): Neighborhood concentrated disadvantage is negatively associated with residents' self-rated health.

### 2.2 The regulatory mechanism: exposure as “dosage”

A key limitation of prior research is its failure to account for the intensity of interactions between actors and their environments. Residents are not static: their daily activities, social engagement, and time allocation determine their functional exposure to the community environment. Chetty et al. (2016) conceptualized this as “dose”—the intensity of environmental impacts absorbed by individuals (21).

This level of exposure acts as a behavioral regulator. For “highly exposed” individuals, those who invest significant time in the community, participate in local governance, or rely on local networks, the community environment becomes a dominant psychological and social force (22). Conversely, for “low-exposure” individuals whose lives are disconnected from their residential area (such as commuters whose social networks lie outside the community), the link between community structure and health may weaken (23). For instance, vulnerable groups such as the older adult or children often have restricted activity spaces, making them more sensitive to local environmental stressors or resources (24). Therefore, exposure that increase community engagement should theoretically amplify the community environment's impact on health—whether active or negative.

Drawing upon Cockerham's Health Lifestyle Theory (25), health behaviors are best understood not as purely individual choices but as structured outcomes shaped by the interplay between social constraints and human agency. In the urban neighborhood context, concentrated disadvantage functions as a structural filter that narrows the range of lifestyle options realistically available to residents.

This filtering operates through two related pathways. Spatially, an individual's daily routines, including community participation and physical exercise, which determine the frequency and intensity of exposure to neighborhood resources or environmental hazards (26). Socially, the degree to which residents participate in local networks shapes their access to psychosocial resources such as social capital and collective efficacy (27). As Kawachi and Berkman (28) have argued, a socially engaged lifestyle fosters a deeply embedded pattern of community exposure that may buffer the chronic stress associated with living in disadvantaged areas (29).

Whether such buffering applies across all urban contexts, however, remains theoretically uncertain. In places with severe residential stratification—such as rapidly urbanizing Chinese cities—neighborhood disadvantage might undermine the social and physical conditions necessary for positive engagement patterns. In these cases, lifestyle behaviors may no longer soften the health impacts of structural deprivation; instead, they may operate on entirely separate paths. Addressing this theoretical issue is a key goal of the present study. We therefore propose Hypothesis 2 (H2): Individual community exposure patterns are independently associated with self-rated health, even after accounting for macro-level neighborhood structural disadvantage.

### 2.3 The social-ecological model and hypothesized pathways

**Figure 1:**
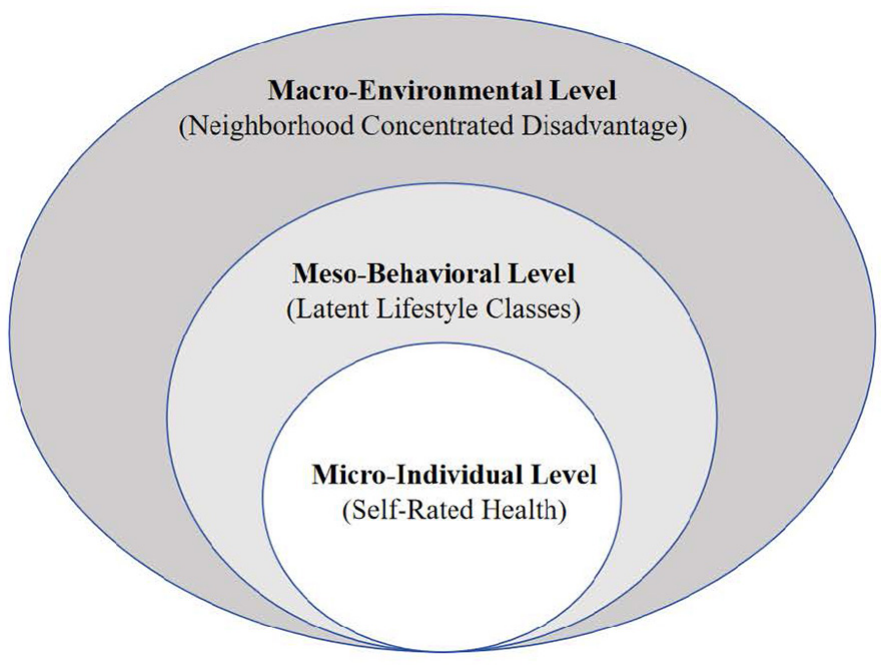
Conceptual framework based on the social-ecological model.

The Social-Ecological Model indicates that health outcomes result from the dynamic interaction between individuals and their nested social environments (30). We base this framework on three pathways through which neighborhood conditions and lifestyle behaviors influence health. The first is a direct structural pathway: concentrated disadvantage limits health from the top down by restricting access to health-supporting resources and exposing residents to ongoing physical and psychosocial stressors, regardless of their individual actions. The second is an individual agency pathway: lifestyle patterns mirror the exercise of agency within structural constraints, and different lifestyle typologies are expected to independently predict health outcomes. The third is an interaction pathway, which is the main empirical focus of this study: we examine whether a healthy lifestyle buffers the negative health effects of neighborhood disadvantage—the traditional buffering hypothesis—or whether structural conditions and behavioral patterns have separate, additive effects on health, as our parallel tracks hypothesis proposes. The conceptual framework is illustrated in Figure 1.

## 3 Materials and methods

### 3.1 Data source and sample selection

The CLDS is widely recognized for analyzing social dynamics in China (31, 32), employing a multi-stage stratified sampling design to collect comprehensive data at the individual, household, and community levels. Although designed as a rotating panel study, this research specifically utilizes cross-sectional data from the 2018 wave. This specific wave was chosen because it provides the most comprehensive measurements of “community participation” and ensures an optimal sample size for integrating Latent Class Analysis (LCA) with multilevel modeling. Geographically, the 2018 sample covers 28 provinces, municipalities, and autonomous regions across China (excluding Hong Kong, Macao, Taiwan, Tibet, Hainan, and Xinjiang).

The analytical sample was limited to urban communities for two reasons. Theoretically, neighborhood disadvantage frameworks are grounded in urban sociology. Empirically, Chinese urban communities and rural villages function under fundamentally different social systems, distinguished by the hukou system. The former is centered on formal, stranger-based governance, while the latter relies on kinship-based networks, making cross-setting comparisons of structural deprivation analytically impossible.

The initial urban subsample included 4,770 individuals from 133 communities. Missing data were handled through sequential listwise deletion: 893 cases were discarded due to missing behavioral indicators for Latent Class Analysis (LCA), and 1,481 were excluded because of missing International Socio-Economic Index (ISEI) scores for occupational status—a known data limitation in the CLDS for self-employed and informal sector workers. To evaluate potential selection bias, we compared the baseline characteristics (age, sex, hukou status, and self-rated health) of respondents included vs. those excluded; no statistically significant differences were found, indicating that ISEI missingness probably did not cause systematic bias (see Supplementary Table S1). Additionally, 264 cases were dropped due to missing covariates, resulting in an analytical sample of 2,132 complete cases. Propensity Score Matching (PSM) then removed 27 off-support cases, resulting in a final sample of 2,105 individuals across 126 communities. A flowchart of the sample selection process is available in Supplementary Figure S1.

To ensure the reliability of community-level aggregated variables, we followed methodological standards that recommend minimizing aggregation bias by ensuring clusters contain sufficient observations and that the intraclass Correlation Coefficient (ICC) exceeds 0.2 (33). In the CLDS, approximately 35 households were randomly sampled per community, and the ICCs for key indicators consistently exceeded this threshold.

### 3.2 Measures

#### 3.2.1 Dependent variable: self-rated health (SRH)

Self-rated health serves as an effective alternative measure of overall physical and mental well-being (34). Respondents assessed their health status using a five-point scale ranging from “very healthy” to “very unhealthy.” Following standard practice in health inequality research, we dichotomized this variable into a binary outcome: 1 = “Healthy” (including “Very Healthy” and “Healthy”), 0 = ‘Unhealthy' (including “Fair,” “Unhealthy,” and “Very Unhealthy”).

#### 3.2.2 Independent variable: neighborhood context (concentrated disadvantage)

To capture the structural constraints of the community system, we constructed a “Concentrated Disadvantage” index. Following the methodology of Lei (35, 36), we utilized four community-level indicators: (1) unemployment rate, (2) poverty rate (based on World Bank standards), (3) proportion of residents with education below high school, and (4) median household income. Principal Component Analysis (PCA) was applied to these indicators to generate a composite score. A single factor with an eigenvalue greater than 1 was extracted, explaining 52.28% of the total variance, with factor loadings ranging from 0.60 to 0.84 (37). A higher score indicates a higher level of structural disadvantage in the neighborhood.

#### 3.2.3 Moderator: community exposure (lifestyles)

To capture the intensity of agent-environment coupling, we operationalized community exposure through five dimensions of community-related lifestyles, which served as observed indicators for the Latent Class Analysis. These indicators comprised two health risk behaviors—specifically, a history of continuous smoking for at least 1 year and a habit of weekly alcohol consumption. Rooted in the environmental stress and coping framework of neighborhood effects, structural disadvantage not only limits access to recreational resources but also serves as a chronic stressor. In disadvantaged or rapidly changing Chinese communities, smoking and drinking are often socialized behaviors used to cope with environmental stressors or as substitutes for formal social participation (22). From this perspective, “community exposure” is more than just spatial presence. It is a behavioral manifestation of how residents navigate their structural environment. By combining social engagement (positive exposure) with substance use (maladaptive coping), our LCA captures a more holistic “exposure profile” that reflects how macro-level deprivation penetrates individual lifestyles. Three dimensions of active engagement: regular physical exercise in the past month; neighborly mutual assistance (dichotomized into frequent vs. infrequent interaction); and community social participation. Crucially, social participation was defined as engaging in at least one of seven types of organizational activities (recreation, sports, senior associations, skill training, knowledge learning, volunteering, or religious groups) strictly within the community boundaries. These five binary variables were collectively analyzed to identify distinct latent typologies of resident-community exposure.

#### 3.2.4 Control variables

We controlled for individual-level sociodemographic characteristics, including age, gender (1 = female), marital status (1 = married), and educational level (categorized into four groups: primary or below, junior high, senior high, and college or above). Socioeconomic status was measured using the International Socio-Economic Index of Occupational Status (ISEI) and annual personal income (log-transformed). Subjective social status was also measured on a 10-point scale (1 = lowest, 10 = highest).

At the community and psychosocial level (38), mental health was assessed using the 20-item Center for Epidemiologic Studies Depression Scale (CES-D-20) (39). To capture urban community structural characteristics (40), we examined two key dimensions: spatial location and institutional factors. Spatial location was categorized as urban area, market town, or suburb, serving as a proxy for differential access to community resources. Hukou status (1 = urban, 0 = rural) was included to capture institutional disparities in public service access. Social support was operationalized as the number of close local contacts available to provide assistance (41). Given that the CLDS 2018 does not include a direct measure of length of residence, the proportion of homeowners within the community was used as a proxy for residential stability (42, 43). Finally, community cultural environment was measured through two items assessing respondents' familiarity with and trust in their neighbors, each rated on a 5-point scale from 1 (very unfamiliar/distrustful) to 5 (very familiar/trustful).

### 3.3 Analytical strategy

All data preparation and statistical analyses were conducted using Stata version 17.0. To rigorously operationalize our variables, control for confounding, and test the proposed socioecological mechanisms, the analytical strategy was executed in three systematic steps: (1) Latent Class Analysis (LCA) to derive community exposure patterns; (2) Propensity Score Matching (PSM) to mitigate selection bias; and (3) a weighted multilevel logistic regression model to estimate the main and interactive effects.

#### 3.3.1 Latent class analysis

Data analysis was conducted in two phases, aiming to capture both behavioral heterogeneity and stratified constraints within the urban system. First, to operationalize community exposure, we employed latent class analysis (LCA) (44). Unlike traditional variable-centering methods that rely on population averages, this person-centered technique identifies latent types based on the intersection of residents' daily behaviors. By grouping individuals into homogeneous subgroups, LCA empirically identifies unique behavioral-environmental coupling patterns that are often obscured by alternative approaches. Second, accounting for the nested data structure, where individuals are embedded within specific community systems, we employed a multilevel logistic regression model (random-intercept model). This multilevel modeling approach addresses violations of independence assumptions inherent in clustered data by decomposing variance in health outcomes across individual and community levels (45). Specifically, the model estimates both the direct contextual effect of community disadvantage (macro-level) on self-rated health and the cross-level interaction effects of identified community exposure types (micro-level), rigorously testing the proposed socioecological moderation mechanism.

#### 3.3.2 Propensity score matching

Given the observational nature of the study, participants were not randomly assigned to communities with varying levels of disadvantage. To address the potential selection bias, PSM was introduced. First, neighborhood concentrated disadvantage was dichotomized into high and low categories based on its mean value. Communities above the mean were defined as the “treatment group” (high disadvantage), while those below served as the “control group.” A logit model was used to estimate the probability (propensity score) of residing in a highly disadvantaged community. Age, marital status, gender, hukou (household registration) status, education level, and the logarithm of annual income were set as baseline confounding variables (Supplementary Table S2).

## 4 Results

### 4.1 Descriptive statistics

Descriptive statistics are presented in Table 1. The mean (SD) age of 2,132 participants was 42.43 (11.14), and 48.4% of the sample were female. Regarding institutional factors, 48.73% of the participants held a rural hukou status. At the community level, the mean proportion of homeowners stood at 0.38. In terms of spatial location, the majority of participants (61.73%) resided in urban areas, followed by those living in suburbs (21.39%) and market towns (16.89%).

**Table 1:** Descriptive statistics of the sample (*N* = 2,132).

Variable	Category/description	Mean/%	SD
Individual level			
Gender	Male	51.6%	–
	Female	48.4%	–
Marital status	Married	82.1%	–
	Unmarried	17.9%	–
Hukou status	Urban	51.27%	
	Rural	48.73%	
Education level	Primary or below	11.68%	–
	Junior high	29.78%	–
	Senior high	24.06%	–
	College or above	34.47%	–
Age	15–83	42.43	11.14
Income (log)	Log (annual income + 1)	9.40	2.72
ISEI	Occupational status index	34.39	23.65
Subjective social status	Scale 1–10	4.90	1.74
CES-D20	0–60	53.18	8.57
Neighbor familiarity	1–5	3.34	0.97
Neighborhood trust	1–5	3.42	0.76
F-owner ratio	0–1	0.38	0.23
Social support network	0-100	10.03	14.63
Location	Urban area	61.73%	
	Market town	16.89%	
	Suburb	21.39 %	
System level			
Neighborhood Disadvantage **Outcome**	Factor score (standardized)	0.00	1.00
Self-rated health	Healthy	70.64%	–
	Unhealthy	29.36%	–

Data sourced from CLDS 2018. ISEI and subjective social status values have been corrected to conform to standard scale ranges.

### 4.2 Latent class analysis of community exposure patterns

To identify distinct patterns of community exposure, Latent Class Analysis (LCA) was conducted using five observed indicators: smoking, drinking, regular exercise, neighborhood participation, and neighborhood reciprocity. Models with one to four latent classes were estimated and compared (Table 2).

**Table 2:** Fit statistics for latent class models (1-−4 classes).

Latent classes	*N*	AIC	BIC	Entropy	Categorical probability
Class1	2,132	13,014	13,042	–	1.00
Class2	2,132	12,622	12,706	0.7112	0.273/0.724
Class3	2,132	12,515	12,611	0.7958	0.112/0.207/0.675
Class4	2,132	12,498	12,617	0.704	0.212/0.511/0.182/0.094

AIC, akaike information criterion; BIC, bayesian information criterion. Lower values indicate better model fit. Categorical probability refers to the estimated class proportions for each latent class solution. Model selection was based on overall model fit, parsimony, and interpretability.

**Figure 2:**
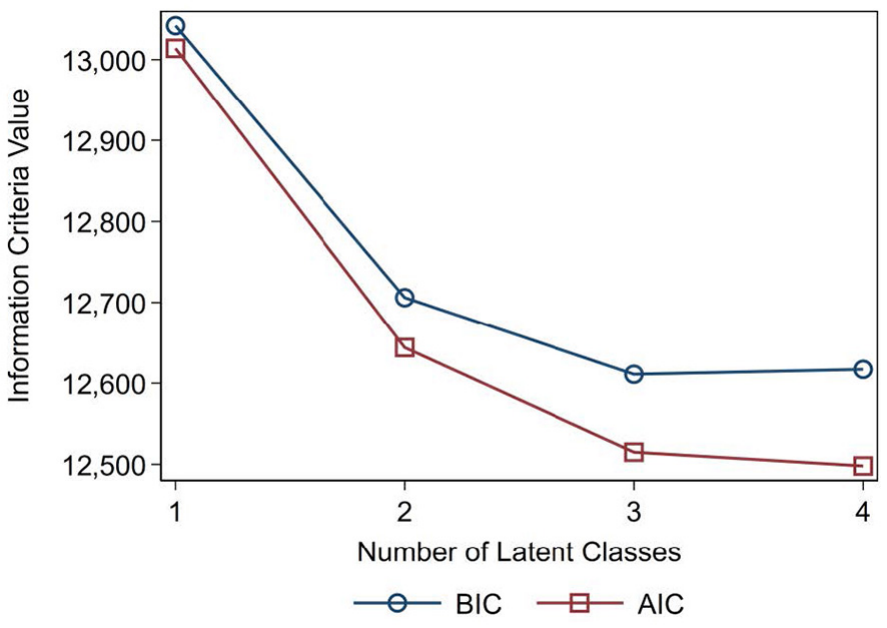
Elbow plot.

Furthermore, to provide a more intuitive visual representation of model selection, we have added an Elbow Plot (Figure 2) depicting the trajectories of AIC and BIC.

To determine the optimal number of latent classes and assess the robustness of the selected solution, comprehensive fit statistics, including AIC, BIC, and Entropy, were evaluated for models with 1 to 4 classes (Table 2). As illustrated in the elbow plot (Figure 2), although the AIC decreased continuously with the addition of classes, the BIC reached its absolute minimum value for the 3-class model (BIC = 12611) before increasing in the 4-class solution (BIC = 12617).

Furthermore, Entropy was used to evaluate the classification quality and class separation. The 3-class model demonstrated the highest entropy value (0.7958), well above the commonly accepted threshold of 0.70 and approaching the ideal threshold of 0.80, indicating excellent classification certainty. In contrast, the entropy values dropped for both the 2-class (0.7112) and 4-class (0.704) models. Based on the convergence of statistical parsimony (minimum BIC), superior classification quality (peak Entropy), and meaningful subgroup proportions, the 3-class model was conclusively selected as the most adequate and robust solution.

Table 3 presents the conditional probabilities of behaviors across the three identified classes. Class 1 was characterized by near-zero probabilities of smoking and drinking, moderate physical exercise (probability = 0.51), and relatively low participation in social organizations (0.28). We labeled this group as the “Health-oriented”. The Class 2 exhibited a paradoxical pattern: while they had high probabilities of smoking (0.86) and drinking (0.86), they concurrently reported the highest probabilities of engaging in physical exercise (0.73) and participating in social organizations (0.96). This suggests a profile of individuals whose substance use may be tied to intense social interactions. Consequently, we designated this group as the “Mixed (Risk/Part)”. The Class 3 represented the most vulnerable demographic, characterized by high probabilities of smoking (0.81) and drinking (0.91), coupled with the lowest probabilities of physical exercise (0.36) and participation in social organizations (0.10). Reflecting their unhealthy behaviors and lack of social integration, we named this group the “High Risk Isolated”.

**Table 3:** Sample distribution and predicted probabilities of community lifestyles.

Indicator	Class1: health-oriented	Class2: mixed (risk/part)	Class3: high risk isolated
Health risk behaviors			
Smoking (Probability of “Yes”)	0.083	0.884	0.847
Drinking (Probability of “Yes”)	0.187	0.895	0.963
System engagement			
Regular exercise (Probability of “Yes”)	0.555	0.849	0.357
Community participation (Probability of “Yes”)	0.306	0.873	0.144
Neighborhood reciprocity (Probability of “Yes”)	0.379	0.519	0.362
Class distribution			
Sample proportion	13.37%	21.11%	65.52%
Sample size (*N*)	285	450	1397

Values represent the conditional probability of engaging in the behavior given class membership. Bold values indicate the defining characteristics of each typology. Source: CLDS (2018).

**Figure 3:**
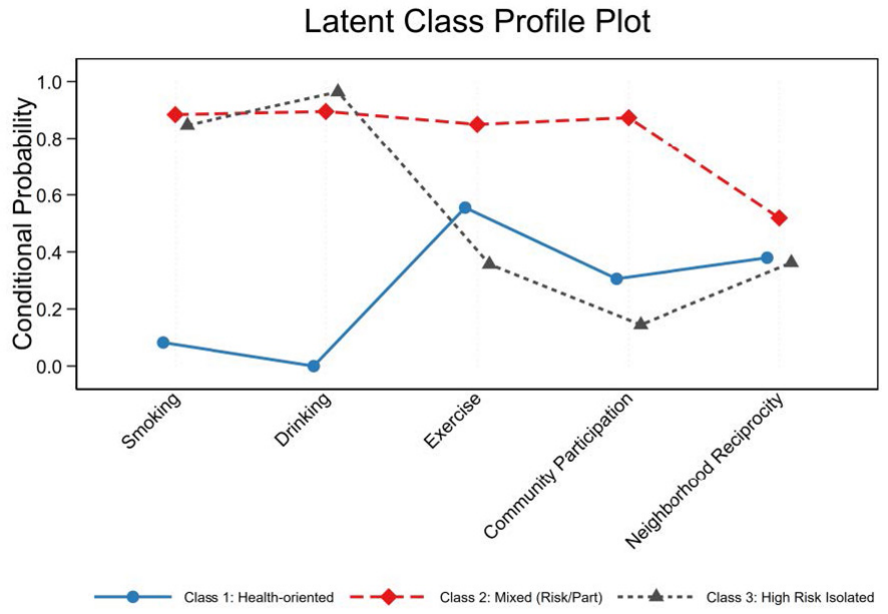
Latent class profile plot.

Figure 3 illustrates the latent class profile plot based on the conditional probabilities of the five indicators across the three identified classes. For the Class 1, positive health behaviors (abstaining from smoking/drinking, moderate exercise) are largely decoupled from community engagement. Their health management is highly individualized and does not rely on intense social networking. The Class 2 presents a fascinating paradox. Their intense social participation acts as a vehicle for both physical activity and risk behaviors. The vulnerable group (Class 3) illustrates how profound social isolation exacerbates risk behaviors. The lack of community participation deprives them of the psychosocial support and peer encouragement needed to initiate healthy routines or cease substance use.

#### 4.2.1 Sociodemographic profiles of community lifestyle types

The three lifestyle groups showed significant sociodemographic differences (Table 4). The health-oriented group was predominantly female, with a mean age of 42.9 years. Their educational attainment, occupational prestige index (ISEI), and subjective social status were below the sample mean, indicating lower socioeconomic status (SES). In contrast, the mixed (risk/part) group represents the community's socioeconomic elite. This group has the highest share of college graduates (47.93%) and reports the highest household income, occupational status, and subjective social status. The high-risk isolated group is predominantly male (97.8%), older (mean age = 44.75 years), and characterized by medium educational attainment and lower occupational status, although household income is comparable to the sample mean.

**Table 4:** Sample distribution and predicted probabilities of community lifestyles (*N* = 2,132).

Variable	Mean/% (SD)	Overall	Class 1: health-oriented	Class 2: mixed (risk/part)	Class 3: high-risk isolated	*P* value
Sample size (*N*)		2,132	285	450	1,397	
Gender	Female (%)	48.41	97.54	45.78	44.09	0.000
	Male (%)	51.59	2.46	54.22	55.91	
Marital status	Married (%)	82.08	85.61	79.56	82.18	0.112
	Unmarried (%)	17.92	14.39	20.44	17.82	
Hukou status	Urban (%)	51.27	51.23	60.44	44.45	0.000
	Rural (%)	48.73	48.77	39.56	55.55	
Age	M (SD)	42.43 (11.14)	44.68 (11.53)	42.06 (11.12)	42.09(11.02)	0.604
Income (log)	M (SD)	9.40 (2.72)	9.51 (1.73)	9.27(3.24)	9.42 (2.70)	0.000
ISEI	M (SD)	34.39 (23.65)	30.59 (20.49)	35.42 (25.90)	34.19 (23.39)	0.000
Subjective social status	M (SD)	4.90 (1.73)	4.48 (1.79)	5.11 (1.82)	4.88 (1.68)	0.067
Education level (%)	Primary or below (%)	11.68	10.53	6.44	13.60	0.000
	Junior high (%)	29.78	33.33	22.67	31.35	
	Senior high (%)	24.06	30.53	22.44	23.26	
	College or above (%)	34.47	25.61	48.44	31.78	
CES-D20	M (SD)	53.18 (8.57)	53.46 (8.30)	53.20 (7.63)	53.11 (8.90)	0.000
Neighbor familiarity	M (SD)	3.34 (0.97)	3.37 (0.98)	3.64 (0.90)	3.24 (0.96)	0.190
Neighborhood trust	M (SD)	3.42 (0.75)	3.42 (0.80)	3.64 (0.68)	3.35 (0.76)	0.005
F-owner ratio	M (SD)	0.38 (0.23)	0.28 (0.21)	0.36 (0.22)	0.38 (0.23)	0.072
Social support network	M (SD)	10.03 (14.63)	12.37 (17.71)	11.19 (15.16)	9.18 (13.66)	0.000
Location	Urban area (%)	63.06	56.49	60.89	63.06	0.025
	Market town (%)	16.89	16.49	20.22	15.89	
	Suburb (%)	21.39	27.02	18.89	21.05	

*P*-values derived from Chi-square tests for categorical variables and ANOVA for continuous variables.

### 4.3 Results of propensity score matching and balance test

#### 4.3.1 Propensity score matching

**Figure 4:**
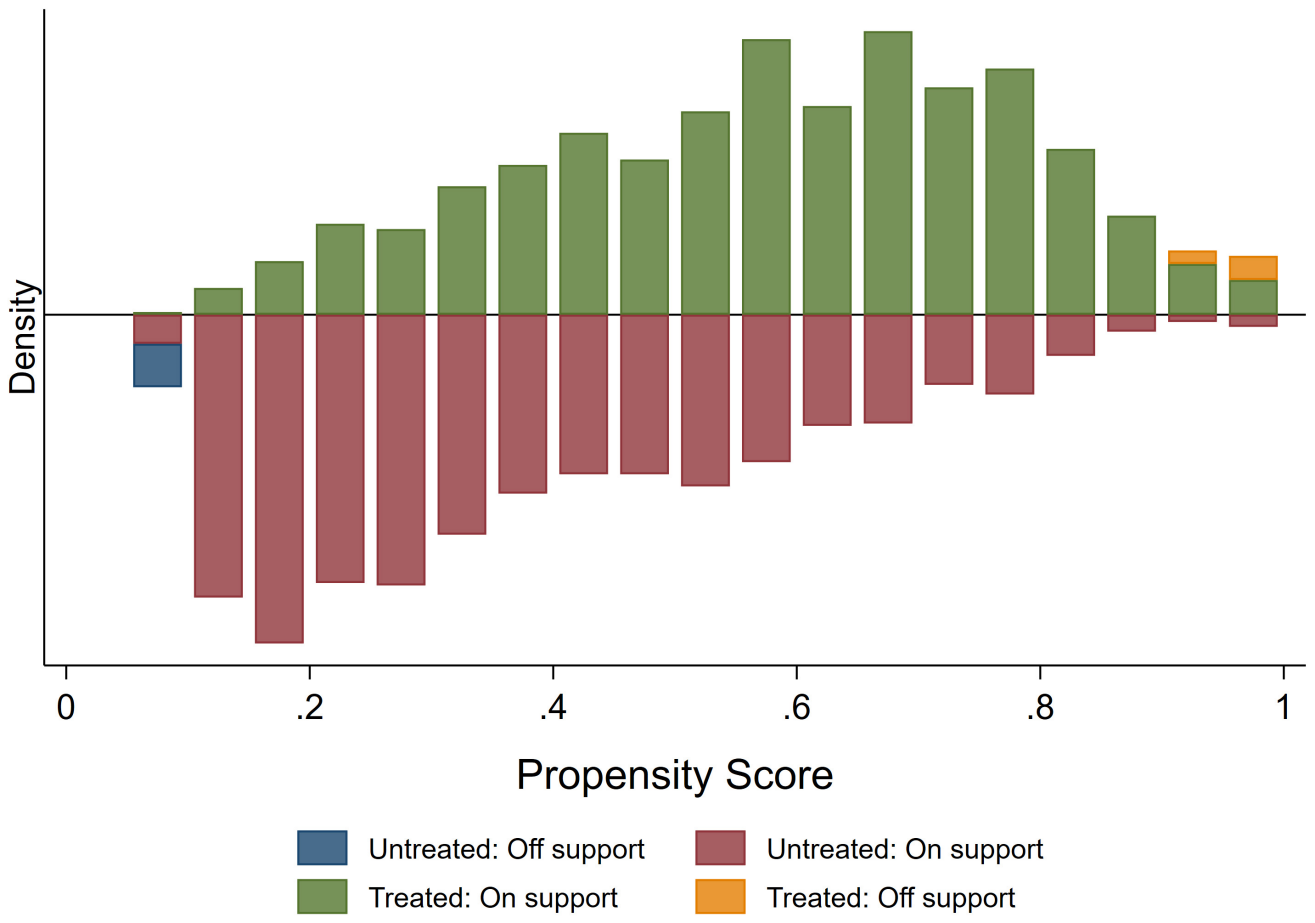
Common support histogram of propensity scores before and after matching.

Neighborhood disadvantage was measured as a binary treatment indicator, with individuals living in communities above the sample mean labeled as the treatment group (high disadvantage = 1) and those below as the control group (low disadvantage = 0). Propensity scores were estimated through logistic regression, and kernel matching was employed to calculate the Average Treatment Effect for the Treated (ATT). A common support condition was applied, leading to the exclusion of 27 off-support cases. Balance diagnostics confirmed sufficient covariate balance between groups after matching (Figure 4).

The matching weights produced by this procedure were used in the multilevel models and applied to the final analytical sample of 2,105 individuals nested within 126 communities. To address the clustered data structure, all multilevel models included a community-level random intercept, and robust standard errors were calculated throughout. This method combines PSM weights to adjust for observable selection bias with multilevel random effects to account for unobserved community-level heterogeneity, thereby enabling valid inference within the nested data structure.

As shown in Figure 4, the difference in propensity scores for residing in disadvantaged communities between the treatment group and the control group became relatively small, and the trends of their kernel density distributions tended to be consistent, with most values ranging from 0.1 to 0.9. This indicates that the samples after propensity score matching have effectively mitigated the selection bias associated with residing in disadvantaged communities. The results are presented in Table 5.

**Table 5:** Sample balance test of propensity score matching.

Variable	Sample	Disadvantage neighborhood (high)	Disadvantage neighborhood (low)	SD %	Deviation reduction %	*T*	*P*
Gender	Before	0.466	0.500	−6.7	44.1	−1.55	0.121
	After	0.462	0.444	3.8		0.84	0.400
Age	Before	44.489	41.478	18.1	65.2	4.18	0.000
	After	43.511	44.21	−6.3		−1.33	0.184
Marital status	Before	0.839	0.803	9.5	68.0	1.24	0.029
	After	0.841	0.830	3.0		0.52	0.483
Hukou	Before	0.370	0.593	−45.6	96.6	−10.51	0.000
	After	0.369	0.361	1.6		0.35	0.724
Education level	Before	2.482	3.110	−63.2	93.0	−14.56	0.000
	After	2.491	2.447	4.4		0.95	0.340
Income (log)	Before	8.777	9.969	−44.3	85.9	−10.36	0.000
	After	8.945	9.132	−6.2		−1.22	0.221
Subjective social status	Before	33.078	33.583	−10.6	43.4	−2.44	0.000
	After	32.949	31.53	6.0		1.43	0.654
ISEI	Before	33.078	35.583	−10.6	43.4	−2.22	0.015
	After	32.949	31.53	6.0		1.43	0.154
Social support network	Before	9.842	12.326	−66.2	97.3	−21.56	0.224
	After	9.852	9.816	1.0		0.29	0.446
CES-D20	Before	8.967	9.834	−33.2	90.2	−1.09	0.275
	After	8.981	9.087	−4.1		−1.02	0.846
Location	Before	0.446	0.546	−20.0	86.0	−6.50	0.000
	After	0.447	0.443	0.8		0.23	0.201
F-owner ratio	Before	0.360	0.393	−14.9	91.0	−3.42	0.000
	After	0.358	0.355	1.3		0.31	0.758
Neighbor familiarity	Before	3.522	3.182	35.7	97.6	8.24	0.000
	After	3.524	3.516	0.8		0.19	0.849
Neighborhood trust	Before	3.513	3.344	22.4	97.8	5.18	0.000
	After	3.516	3.510	−0.5		−0.11	0.914

Propensity score matching was used to balance baseline characteristics between disadvantaged and non-disadvantaged neighborhoods. Standardized mean differences (percent) below 10% indicate adequate covariate balance. SD denotes the standardized mean difference (%), and Deviation reduction (%) denotes the decrease in standardized bias after matching. *T* and *P* values are from two-sample *t* tests. ISEI denotes the International Socio-Economic Index of Occupational Status. Income is log-transformed.

#### 4.3.2 Balance test

Balance diagnostics represent a critical post-matching phase designed to confirm the comparable distribution of covariates between treatment and control cohorts. Table 5 details the outcomes of the balance assessment obtained via kernel matching, where self-rated health serves as the outcome measure.

As presented in Table 5, before matching, significant differences existed across all variables except for gender, social support network and CES-D20, indicating the presence of selection bias. After matching, there was no significant difference among all variables; among these, the absolute values of the bias for years of education and per capita annual household income decreased by more than 90%. This demonstrates that the matching achieved favorable results and passed the balance test. Furthermore, to ensure the robustness of our findings, K-nearest neighbor matching and radius matching were additionally employed. All three matching approaches yielded highly consistent results, confirming that concentrated neighborhood disadvantage significantly diminishes residents' self-rated health. The detailed results of the alternative matching methods are provided in Supplementary Table S1.

### 4.4 Multilevel logit regression analysis

Multilevel logistic regression models were used to examine how neighborhood disadvantage affects self-rated health and how community exposure moderates this relationship (Table 6). Model 1 shows the basic structural constraint: residents in highly disadvantaged neighborhoods have notably lower odds of reporting good health compared to those in less disadvantaged neighborhoods (β = −0.119, *p* < 0.001). This negative impact remains strong in model 3 (β = −0.125, *p* < 0.001) even after controlling for individual SES and lifestyle factors. The community-level variance stayed relatively stable (slightly increasing from.593 to.598) after considering exposure typology.

**Table 6:** Multilevel logit regression analysis of neighborhood disadvantage, exposure, and self-rated health.

Variables	Model 1	Model 2	Model 3	Model 4
Neighborhood level (L2)				
Concentrated disadvantage	−0.119^***^		−0.125^***^	−0.143^***^
	(0.034)		(0.035)	(0.043)
Individual level (L1)				
Exposure typology (Ref: high risk isolated*)*				
Health-oriented		−0.194^**^	−0.196^**^	−0.204^**^
		(0.069)	(0.069)	(0.069)
Mixed (risk/part)		−0.129^+^	−0.134^+^	−0.137^+^
		(0.250)	(0.252)	(0.248)
Controls				
Gender (female)	−0.168	−0.210	−0.215	−0.211
	(0.161)	(0.171)	(0.172)	(0.172)
Age	−0.047^***^	−0.047^***^	−0.047^***^	−0.047^***^
	(0.010)	(0.010)	(0.010)	(0.010)
Marital status (married)	0.219 (0.275)	0.214 (0.274)	0.220 (0.274)	0.218 (0.274)
Hukou (urban)	−0.227 (0.205)	−0.228 (0.201)	−0.210 (0.201)	−0.203 (0.202)
Education (Ref: primary)				
Junior high	0.142 (0.212)	0.164 (0.215)	0.172 (0.216)	0.176 (0.217)
Senior high	−0.097 (0.247)	−0.060 (0.251)	−0.051 (0.250)	−0.039 (0.252)
College or above	−0.327 (0.249)	−0.223 (0.246)	−0.225 (0.245)	−0.223 (0.246)
Income(log)	−0.008 (0.041)	−0.001 (0.045)	−0.010 (0.042)	−0.010 (0.042)
Subjective social status	0.217^**^ (0.071)	0.209^**^ (0.065)	0.209^**^ (0.065)	0.210^**^ (0.065)
Occupational status (ISEI)	0.007^*^ (0.003)	0.006^+^ (0.003)	0.006^*^ (0.003)	0.006^+^ (0.003)
Social support network	0.006 (0.006)	0.006 (0.006)	0.006 (0.006)	0.006 (0.006)
CES-D20	0.049^***^ (0.011)	0.048^***^ (0.011)	0.048^***^ (0.011)	0.049^***^ (0.011)
Location (Ref: urban area)				
market town	0.067 (0.351)	−0.047 (0.347)	0.096 (0.351)	0.093 (0.351)
Suburb	−0.185 (0.224)	−0.215 (0.229)	−0.183 (0.225)	−0.182 (0.225)
F-owner ratio	0.233	0.274	0.227	0.266
	(0.417)	(0.428)	(0.413)	(0.415)
Neighbor familiarity	−0.028 (0.114)	0.001 (0.111)	−0.005 (0.112)	−0.004 (0.112)
Neighborhood trust	0.226^+^ (0.120)	0.235^+^ (0.122)	0.233^+^ (0.123)	0.229^+^ (0.122)
Cross-level interaction				
Disadvantage × health-oriented				0.259 (0.319)
Disadvantage × mixed (risk/part)				0.311 (0.246)
Constant	−1.344^+^ (0.788)	−1.366^+^ (0.830)	−1.266 (0.822)	−1.308 (0.827)
Random effects				
Intercept variance	0.592^***^ (0.162)	0.627^***^ (0.171)	0.598^***^ (0.168)	0.598^***^ (0.166)
ICC	0.1525	0.1600	0.1538	0.1539
Log pseudolikelihood	−1,093.1	−1,089.6	−1,088.1	−1,086.7
Number of observations	2,105	2,105	2,105	2,105
Number of clusters (communities)	126	126	126	126

Unstandardized coefficients reported with standard errors in parentheses. Significance levels: ^+^ *P* < 0.1,

^*^P < 0.05,

^**^P < 0.01,

^***^P < 0.001.

Model 4 included cross-level interaction terms to examine whether lifestyle class influenced the relationship between neighborhood disadvantage and self-rated health. Neither interaction term was statistically significant (mixed risk/participation × disadvantage: β = 0.259, *p* > 0.05; high-risk isolated × disadvantage: β = 0.311, *p* > 0.05), indicating that the link between neighborhood disadvantage and health remains consistent across various lifestyle classes. These results support the Parallel Tracks Hypothesis (H2): structural disadvantage and individual lifestyle seem to function as independent, additive factors affecting health rather than as a buffering system. Three robustness checks confirmed this finding: an ordinal multilevel model for self-rated health (Supplementary Table S3), reclassification of neighborhood disadvantage into four categories (Supplementary Table S4), and regression analyses utilizing multiple imputation (Supplementary Table S5).

## 5 Discussion

This study examined how neighborhood concentrated disadvantage and individual lifestyle-based community exposure jointly shape self-rated health among urban residents in China, yielding three substantive findings that we discuss in turn.

First, regarding agent heterogeneity, our latent class analysis (LCA) identified three distinct patterns of agent-environment coupling: health-oriented, mixed (risk/part), and high risk isolated. These typologies reflect the diversity of how residents navigate their community systems. The health-oriented class maintained positive health behaviors, including abstinence from smoking and drinking and moderate physical exercise, while reporting comparatively low engagement in formal social organizations. This pattern suggests a self-directed rather than community-embedded approach to health management. As shown in Table 4, the group is almost exclusively female (97.54%), with compounded socioeconomic disadvantages and low homeownership rates, consistent with backgrounds of rural-to-urban migration or precarious urban residency. Many older female migrants are absorbed in informal labor or intergenerational caregiving responsibilities, which limits both the time and institutional access required for formal community participation (11). Many older female migrants in Chinese cities are either absorbed in informal, low-tier labor markets or burdened with heavy intergenerational caregiving responsibilities (e.g., older adult drifter caring for grandchildren). Consequently, they lack the cultural capital, discretionary time, and institutional resources required to engage in formal urban social organizations or structured leisure activities.

The mixed risk/participation class presented a seemingly paradoxical profile, combining the highest levels of community participation and physical exercise with equally high rates of smoking and drinking. This pattern likely reflects the cultural embeddedness of socializing in Chinese urban settings, where community networking frequently involves collective physical activities alongside social gatherings in which substance use serves as a social lubricant (8). For this group, risk behaviors are socially integrated practices rather than isolated personal choices, and health outcomes reflect a tension between the benefits of strong social engagement and the costs of regular substance exposure. The high-risk isolated class, by contrast, illustrates how social marginalization and health-risk behaviors can reinforce one another. The near-absence of community participation in this group coincides with high rates of substance use and physical inactivity. Without access to the psychosocial support and behavioral norms that community networks provide, health-risk behaviors tend to persist and accumulate, placing this group in the most structurally vulnerable position in the sample.

Second, consistent with extensive evidence on neighborhood effects (46, 47), our multilevel analysis confirmed that higher levels of neighborhood concentrated disadvantage were associated with poorer self-rated health, independent of individual socioeconomic characteristics. This finding reinforces the view that neighborhood socioeconomic conditions constitute a structural health risk that constrains residents' health opportunities beyond individual attributes (48). In disadvantaged communities, limited access to health-supportive infrastructure and greater exposure to environmental stressors may jointly contribute to adverse health outcomes (20). In further analyses, we categorized the continuous neighborhood concentrated disadvantage score into four quartiles. The results revealed that only the fourth quartile (representing the highest level of neighborhood disadvantage) exerted a significant negative effect on health outcomes. This finding indicates a clear threshold effect regarding the impact of neighborhood deprivation.

A central contribution of this study is to examine whether lifestyle-based community exposure modifies the health impact of neighborhood disadvantage. Contrary to our initial hypothesis that higher exposure might amplify environmental health risks (49), we observed no buffering effect. Our findings reveal that residents in disadvantaged neighborhoods face a “double jeopardy”. They are penalized once by their environment and potentially again by their lifestyle choices, but these two factors operate on parallel tracks. A central contribution of this study is to test whether lifestyle-based community exposure moderates the health impact of neighborhood disadvantage. We found no evidence of a significant cross-level interaction: neither lifestyle class moderated the association between neighborhood disadvantage and health. Although prior research has proposed that community participation and physical activity may buffer the adverse effects of deprived environments (50, 51), our results do not support this buffering mechanism in the contemporary Chinese urban context. Rather, the data are consistent with an additive model: residents in disadvantaged neighborhoods who also exhibit high-risk lifestyle patterns accumulate two independent health penalties. This underscores that individual behavioral efforts alone are insufficient to offset the structural harm of living in severely deprived communities.

### 5.1 Implications

These findings have two practical implications for urban public health policy. At the structural level, because neighborhood disadvantage affects health independently of individual behavior, encouraging residents to adopt healthier lifestyles, while worthwhile, is not sufficient to close health disparities in deprived areas. Meaningful progress requires upstream, neighborhood-level action: improving housing conditions, ensuring safe and accessible public spaces, and expanding local economic opportunities. Placing the primary burden of health improvement on already-disadvantaged individuals is neither equitable nor effective.

At the individual level, the three lifestyle classes identified in this study suggest different intervention priorities. For the health-oriented class, whose healthy behaviors coexist with low social engagement, low-key activity-based programs such as community sports clubs or neighborhood walking groups may help build social connections without the demands of more intensive socializing. For the mixed risk/participation class, the challenge is to preserve their high levels of community engagement while reducing exposure to substance use; creating smoke- and alcohol-free social spaces offers one practical avenue. For the high-risk isolated class, standard health promotion campaigns are unlikely to be effective given their limited community engagement. More proactive strategies, such as social prescribing in which clinicians refer patients to community activities as part of routine care, or the deployment of community health navigators to reach isolated residents directly, are more likely to prove useful for this group.

### 5.2 Limitations

Despite utilizing a large-scale national dataset and advanced modeling techniques, this study has several limitations. First, the cross-sectional design of the China Labor-force Dynamics Survey (CLDS) prevents definitive causal conclusions between lifestyles and health. Future longitudinal studies are necessary to observe changes over time. Second, by focusing solely on urban communities, the results may not apply to rural populations. As sociological research emphasizes, rural residents display notably different lifestyle patterns and social engagement. Despite thorough multilevel adjustments, unobservable factors like genetic susceptibility or length of residence might still impact the outcomes results. Furthermore, China's administratively defined “community” concept may differ from residents' “perceived neighborhood boundaries” (52), potentially introducing spatial measurement errors. Future studies should employ finer-grained geospatial data to define activity spaces more continuously. Third, while the null interaction result can be interpreted as supporting the Parallel Tracks Hypothesis in theory, this interpretation should be approached with caution. A lack of statistical significance does not prove independence: the study might lack the statistical power to detect small interactions, and using a binary disadvantage indicator may weaken the estimates. Future research with continuous disadvantage measures, larger samples, and longitudinal designs should try to replicate and clarify this finding.

## 6 Conclusions

This study identified three distinct lifestyle typologies among urban residents: the health-oriented class, the mixed risk/participation class, and the high-risk isolated class. Our findings show that both neighborhood concentrated disadvantage and individual lifestyle typologies have significant and independent effects on self-rated health. Contrary to the traditional buffering hypothesis, we found no significant interaction between neighborhood conditions and lifestyle behaviors.

These results support the idea that neighborhood disadvantage and individual lifestyle operate on two “Parallel Tracks” within modern Chinese urban spaces. This suggests that while a healthy lifestyle provides clear individual benefits, it cannot structurally counteract or lessen the strict health penalties caused by severely deprived spatial environments. Residents in these areas face a “double jeopardy”: they are penalized once by their deprived surroundings and possibly again by their lifestyle choices, but these two risks do not overlap to offer relief. Achieving health equity requires more than just individual lifestyle changes; it demands significant structural reforms that address the spatial roots of urban disadvantage.

## Data Availability

The original contributions presented in the study are included in the article/Supplementary material, further inquiries can be directed to the corresponding authors.
