# Supplementary material for: Lifestyle-based community exposure and self-rated health: a multilevel analysis of neighborhood disadvantage in Urban China
Source: Front Public Health. 2026 Apr 10;14:1785410. doi: 10.3389/fpubh.2026.1785410 (PMC13106475; doi:10.3389/fpubh.2026.1785410)
Supplement: Supplementary file 1 [file Supplementary_file_1.doc]

Respondents included in analysis n=2105

Total number of 4770 respondents

Respondents

n=3877

Respondents

n=2396

Missing data n=264,

unmatched n= 27

ISEI missing

n=1481

Respondents(LCA) missing n=893

**Figure S1.** Flowchart of the study population

**Table S1.** Propensity Score Matching Estimation Results (after matching)

| **Matching methods** | **Treat group** | **Control group** | **ATT** | **Standard Error** | **T** |
| --- | --- | --- | --- | --- | --- |
| K nearest-neighbor matching | 1.003 | 1.102 | -0.208 | 0.050 | -4.16*** |
| Radius cal | 998 | 1.102 | -0.157 | 0.048 | -3.29*** |
| Kernal matching | 1.003 | 1.102 | -0.170 | 0.045 | -3.76*** |

*Notes:*K-nearest neighbor matching, K=4, Radius matching, the radius is 0.01.

**Table S2.** Determinants of residing in a disadvantaged community: results from logistic regression

| Variables | **Logistic Model** |
| --- | --- |
| Female | -0.186^+^ |
|  | (0.099) |
| Age | -0.007 |
|  | (0.005) |
| Married | -0.118 |
|  | (0.142) |
| Urban hukou | -0.659^***^ |
|  | (0.104) |
| Education | -0.517^***^ |
|  | (0.059) |
| Income(log) | -0.257^***^ |
|  | (0.028) |
| Subjective Social Status | -0.045 |
|  | (0.029) |
| Occupational Status (ISEI) | 0.003 |
|  | (0.002) |
| Social Support Network | -0.003 |
|  | (0.004) |
| CES-D20 | -0.001 |
|  | (0.006) |
| Location | 0.467^***^ |
|  | (0.060) |
| F-owner ratio | 0.378^+^ |
|  | (0.227) |
| Neighbor Familiarity | 0.293^***^ |
|  | (0.066) |
| Neighborhood Trust | 0.030 |
|  | (0.084) |
| Constant | 2.782^***^ |
|  | (0.546) |
| Sample Size | 2132 |
| Pseudo R^2^ | 0.1656 |

*Notes:* ^+^ *p* < 0.1, ^*^ *p* < 0.05, ^**^ *p* < 0.01, ^***^ *p* < 0.001

**Table S3.** Multilevel logit regression analysis of neighborhood disadvantage, exposure, and self-rated health (N=2105)

| Variables | Model 1 | Model 2 | Model 3 | Model 4 |
| --- | --- | --- | --- | --- |
| Concentrated Disadvantage | -0.300^**^ |  | -0.321^**^ | -0.378^**^ |
|  | (0.096) |  | (0.099) | (0.128) |
| Gender (Female) | -0.127 | -0.213 | -0.219^+^ | -0.219^+^ |
|  | (0.135) | (0.131) | (0.132) | (0.131) |
| Age | -0.042^***^ | -0.042^***^ | -0.042^***^ | -0.042^***^ |
|  | (0.008) | (0.008) | (0.008) | (0.008) |
| Marital Status (Married) | 0.379 | 0.370 | 0.373 | 0.377 |
|  | (0.248) | (0.247) | (0.245) | (0.244) |
| Hukou (Urban) | -0.045 | -0.063 | -0.042 | -0.040 |
|  | (0.152) | (0.152) | (0.150) | (0.151) |
| Education (Ref: Primary) |  |  |  |  |
| Junior High | 0.095 | 0.109 | 0.114 | 0.115 |
|  | (0.232) | (0.234) | (0.235) | (0.233) |
| Senior High | -0.036 | 0.004 | 0.009 | 0.015 |
|  | (0.243) | (0.248) | (0.247) | (0.248) |
| College or above | -0.226 | -0.145 | -0.150 | -0.145 |
|  | (0.263) | (0.273) | (0.272) | (0.275) |
| Income(log) | -0.002 | 0.010 | -0.003 | -0.003 |
|  | (0.031) | (0.037) | (0.031) | (0.031) |
| Subjective Social Status | 0.177^**^ | 0.173^**^ | 0.173^**^ | 0.174^**^ |
|  | (0.062) | (0.060) | (0.059) | (0.059) |
| Occupational Status (ISEI) | 0.010^***^ | 0.010^***^ | 0.010^***^ | 0.010^***^ |
|  | (0.003) | (0.003) | (0.003) | (0.003) |
| Social Support Network | -0.002 | -0.002 | -0.002 | -0.002 |
|  | (0.004) | (0.004) | (0.004) | (0.004) |
| CES-D20 | 0.052^***^ | 0.051^***^ | 0.051^***^ | 0.052^***^ |
|  | (0.011) | (0.011) | (0.011) | (0.011) |
| Location (Ref: Urban Area) |  |  |  |  |
| Market Town | -0.044 | -0.223 | -0.018 | -0.015 |
|  | (0.326) | (0.335) | (0.324) | (0.325) |
| Suburb | -0.414^*^ | -0.474^*^ | -0.412^*^ | -0.413^*^ |
|  | (0.188) | (0.199) | (0.194) | (0.194) |
| F-owner ratio | -0.230 | -0.173 | -0.237 | -0.226 |
|  | (0.404) | (0.428) | (0.404) | (0.406) |
| Neighbor Familiarity | 0.006 | 0.036 | 0.030 | 0.030 |
|  | (0.084) | (0.082) | (0.082) | (0.082) |
| Neighborhood Trust | 0.234^*^ | 0.243^*^ | 0.239^*^ | 0.237^*^ |
|  | (0.103) | (0.103) | (0.104) | (0.103) |
| Exposure Typology (Ref: Health-oriented) |  |  |  |  |
| Mixed (Risk/Part) |  | -0.538^**^ | -0.543^**^ | -0.566^**^ |
|  |  | (0.179) | (0.178) | (0.179) |
| High Risk Isolated |  | -0.421^*^ | -0.430^*^ | -0.442^*^ |
| Cross-level Interaction |  |  |  |  |
| Disadvantage × Health-oriented |  |  |  | 0.184 |
|  |  |  |  | (0.283) |
| Disadvantage × Mixed (Risk/Part) |  |  |  | 0.162 |
|  |  |  |  | (0.221) |
| / |  |  |  |  |
| cut1 | -2.590^**^ | -2.587^**^ | -2.729^**^ | -2.701^**^ |
|  | (0.880) | (0.900) | (0.885) | (0.883) |
| cut2 | -0.296 | -0.295 | -0.439 | -0.411 |
|  | (0.834) | (0.857) | (0.840) | (0.839) |
| cut3 | 1.798^*^ | 1.803^*^ | 1.659^+^ | 1.688^+^ |
|  | (0.853) | (0.882) | (0.867) | (0.870) |
| cut4 | 4.397^***^ | 4.427^***^ | 4.282^***^ | 4.313^***^ |
|  | (0.836) | (0.869) | (0.854) | (0.859) |
| Var (cons [CID2018]) | 0.635^***^ | 0.719^***^ | 0.648^***^ | 0.650^***^ |
|  | (0.168) | (0.174) | (0.170) | (0.171) |

*Notes:* ^+^ *p* < 0.1, ^*^ p < 0.05, ^**^ *p* < 0.01, ^***^ p < 0.001

**Table S4.** Concentrated disadvantage alternative cut-points

| Variables | Model 1 | Model 2 | Model 3 | Model 4 |
| --- | --- | --- | --- | --- |
| (Ref: Disadvantage: Q1) |  |  |  |  |
| Disadvantage: Q2 | -0.156 |  | -0.159 | 0.775+ |
|  | (0.245) |  | (0.249) | (0.459) |
| Disadvantage: Q3 | -0.218 |  | -0.231 | 0.348 |
|  | (0.263) |  | (0.262) | (0.521) |
| Disadvantage: Q4 | -0.702^**^ |  | -0.731^**^ | -0.102 |
|  | (0.259) |  | (0.266) | (0.570) |
| Gender (Female) | -0.125 | -0.213 | -0.216 | -0.209 |
|  | (0.135) | (0.131) | (0.131) | (0.130) |
| Age | -0.042^***^ | -0.042^***^ | -0.042^***^ | -0.040^***^ |
|  | (0.008) | (0.008) | (0.008) | (0.008) |
| Marital Status (Married) | 0.373 | 0.370 | 0.368 | 0.362 |
|  | (0.248) | (0.247) | (0.246) | (0.245) |
| Hukou (Urban) | -0.039 | -0.063 | -0.036 | -0.027 |
|  | (0.152) | (0.152) | (0.150) | (0.149) |
| Education (Ref: Primary) |  |  |  |  |
| Junior High | 0.098 | 0.109 | 0.118 | 0.128 |
|  | (0.234) | (0.234) | (0.236) | (0.238) |
| Senior High | -0.035 | 0.004 | 0.011 | 0.019 |
|  | (0.246) | (0.248) | (0.250) | (0.253) |
| College or above | -0.226 | -0.145 | -0.149 | -0.131 |
|  | (0.265) | (0.273) | (0.274) | (0.276) |
| Income(log) | -0.005 | 0.010 | -0.005 | -0.008 |
|  | (0.030) | (0.037) | (0.030) | (0.031) |
| Subjective Social Status | 0.177^**^ | 0.173^**^ | 0.173^**^ | 0.172^**^ |
|  | (0.062) | (0.060) | (0.059) | (0.057) |
| Occupational Status (ISEI) | 0.010^***^ | 0.010^***^ | 0.010^***^ | 0.010^***^ |
|  | (0.003) | (0.003) | (0.003) | (0.003) |
| Social Support Network | -0.002 | -0.002 | -0.002 | -0.003 |
|  | (0.004) | (0.004) | (0.004) | (0.004) |
| CES-D20 | 0.052^***^ | 0.051^***^ | 0.051^***^ | 0.052^***^ |
|  | (0.011) | (0.011) | (0.011) | (0.012) |
| Location (Ref: Urban Area) |  |  |  |  |
| Market Town | -0.069 | -0.223 | -0.049 | -0.055 |
|  | (0.314) | (0.335) | (0.313) | (0.325) |
| Suburb | -0.419^*^ | -0.474^*^ | -0.420^*^ | -0.406^*^ |
|  | (0.193) | (0.199) | (0.199) | (0.203) |
| F-owner ratio | -0.269 | -0.173 | -0.277 | -0.226 |
|  | (0.397) | (0.428) | (0.397) | (0.398) |
| Neighbor Familiarity | 0.008 | 0.036 | 0.033 | 0.025 |
|  | (0.083) | (0.082) | (0.082) | (0.084) |
| Neighborhood Trust | 0.237^*^ | 0.243^*^ | 0.242^*^ | 0.240^*^ |
|  | (0.103) | (0.103) | (0.104) | (0.106) |
| Exposure Typology (Ref: Health-oriented) |  |  |  |  |
| Mixed (Risk/Part) |  | -0.117 | -0.104 | -1.093 |
|  |  | (0.232) | (0.232) | (0.461) |
| High Risk Isolated |  | -0.538^**^ | -0.535^**^ | -1.161^**^ |
|  |  | (0.179) | (0.178) | (0.432) |
| Cross-level Interaction |  |  |  |  |
| Disadvantage: Q2#Mixed (Risk/Part) |  |  |  | -1.599 |
|  |  |  |  | (0.584) |
| Disadvantage: Q2#High Risk Isolated |  |  |  | -0.920^+^ |
|  |  |  |  | (0.489) |
| Disadvantage: Q3#Mixed (Risk/Part) |  |  |  | -0.967 |
|  |  |  |  | (0.610) |
| Disadvantage: Q3#High Risk Isolated |  |  |  | -0.561 |
|  |  |  |  | (0.531) |
| Disadvantage: Q4#Mixed (Risk/Part) |  |  |  | -0.692 |
|  |  |  |  | (0.735) |
| Disadvantage: Q4#High Risk Isolated |  |  |  | -0.692 |
|  |  |  |  | (0.698) |
| / |  |  |  |  |
| cut1 | -2.850^**^ | -2.049^*^ | -2.457^**^ | -1.820^+^ |
|  | (0.935) | (0.909) | (0.946) | (1.081) |
| cut2 | -0.555 | 0.243 | -0.167 | 0.474 |
|  | (0.884) | (0.866) | (0.896) | (1.050) |
| cut3 | 1.538^+^ | 2.342^**^ | 1.932^*^ | 2.580^*^ |
|  | (0.897) | (0.876) | (0.902) | (1.080) |
| cut4 | 4.136^***^ | 4.965^***^ | 4.554^***^ | 5.214^***^ |
|  | (0.878) | (0.857) | (0.882) | (1.073) |
| Var (cons [CID2018]) | 0.626^***^ | 0.719^***^ | 0.642^***^ | 0.646^***^ |
|  | (0.158) | (0.174) | (0.159) | (0.160) |

*Notes:* ^+^ *p* < 0.1, ^*^ *p* < 0.05, ^**^ *p* < 0.01, ^***^ *p* < 0.001

**Table S5.** Logistic Regression Results on Health Status Using Multiple Imputed Data

| Variables | **Model1** | **Model2** |
| --- | --- | --- |
| Concentrated Disadvantage | -0.233^**^ |  |
|  | (0.072) |  |
| Gender (Female) | -0.135^*^ | -0.268^***^ |
|  | (0.061) | (0.075) |
| Age | -0.044^***^ | -0.041^***^ |
|  | (0.004) | (0.004) |
| Marital Status (Married) | 0.120 | 0.132 |
|  | (0.115) | (0.116) |
| Hukou(Urban) | -0.147 | -0.122 |
|  | (0.101) | (0.105) |
| Education (Ref: Primary) |  |  |
| Junior High | 0.030 | 0.096 |
|  | (0.127) | (0.134) |
| Senior High | 0.034 | 0.153 |
|  | (0.143) | (0.145) |
| College or above | 0.038 | 0.243 |
|  | (0.162) | (0.161) |
| Income(log) | 0.038^+^ | 0.055^*^ |
|  | (0.021) | (0.024) |
| Subjective Social Status | 0.171^***^ | 0.173^***^ |
|  | (0.025) | (0.025) |
| Occupational Status (ISEI) | 0.003 | 0.002 |
|  | (0.002) | (0.002) |
| Social Support Network | 0.004 | 0.004 |
|  | (0.003) | (0.003) |
| CES-D20 | 0.045^***^ | 0.045^***^ |
|  | (0.006) | (0.006) |
| Location (Ref: Urban Area) |  |  |
| Market Town | 0.193 | 0.047 |
|  | (0.236) | (0.233) |
| Suburb | -0.214 | -0.281 |
|  | (0.194) | (0.198) |
| F-owner ratio | 0.218 | 0.209 |
|  | (0.241) | (0.261) |
| Neighbor Familiarity | 0.009 | -0.051 |
|  | (0.048) | (0.054) |
| Neighborhood Trust | 0.098 | 0.070 |
|  | (0.062) | (0.063) |
| Exposure Typology (Ref: Health-oriented) |  |  |
| Mixed (Risk/Part) |  | -0.538** |
|  |  | (0.179) |
| High Risk Isolated |  | -0.421* |
|  |  | (0.209) |
| Constant | -1.356^***^ | -1.660^***^ |
|  | (0.387) | (0.421) |
| Sample size | 4296 | 4296 |
| Pseudo R^2^ | 0.1077 | 0.1032 |

*Notes:* + *p* < 0.1, * *p* < 0.05, ** *p* < 0.01, *** *p* < 0.001. Model 2 presents the Latent Class Analysis (LCA) results based on the first completed dataset, extracted after 30 multiple imputations.
